# Supplementary figures and images for: Transcriptome Analysis Reveals Novel Genes Potentially Involved in Tuberization in Potato
Source: Plants (Basel). 2024 Mar 11;13(6):795. doi: 10.3390/plants13060795 (PMC10975680; doi:10.3390/plants13060795)

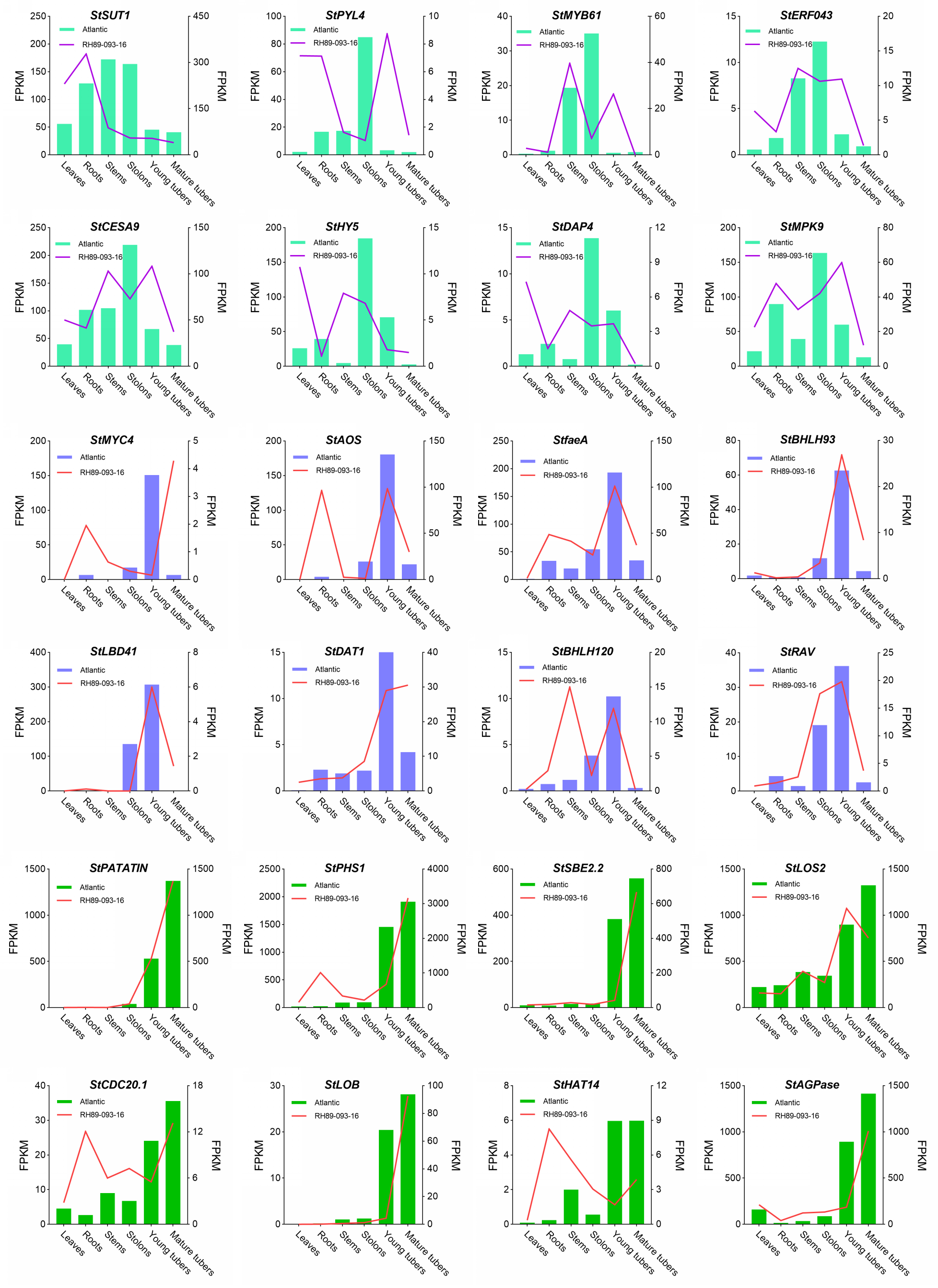

Supplement: Supplementary file 1 [file plants-13-00795-s001.zip › Supplementary Figure S1.tif]
